# The Toxic Effects of Sulfoxaflor Induced in Earthworms (*Eisenia fetida*) under Effective Concentrations

**DOI:** 10.3390/ijerph17051740

**Published:** 2020-03-07

**Authors:** Xiaolian Zhang, Xiuguo Wang, Yalei Liu, Kuan Fang, Tong Liu

**Affiliations:** Plant Protection Research Center, Tobacco Research Institute of Chinese Academy of Agricultural Sciences (CAAS), Qingdao 266101, China; zhangxiaolian04@163.com (X.Z.); wangxiuguo@caas.cn (X.W.); 15621033836@163.com (Y.L.); fk19970312@163.com (K.F.)

**Keywords:** HPLC-MS/MS, effective concentrations, OH^−^, 8-OHdG, neonicotinoid insecticides

## Abstract

Sulfoxaflor is a new kind of neonicotinoid insecticide that is used to control sap-feeding insect pests. In this study, a hazard assessment of sulfoxaflor on soil invertebrate earthworms was performed under effective concentrations. The results showed that different exposure times and doses had significant influence on the toxicity of sulfoxaflor. Sulfoxaflor degraded quickly in artificial soil with a degradation rate of 0.002–0.017 mg/(kg·d) and a half-life of 12.0–15.4 d. At 0.5 mg/kg and 1.0 mg/kg, the ·OH^−^ content, antioxidant enzyme activeities, thiobarbituric acid reactive substances (TBARS) content and 8-OHdG content had significant differences compared to those in the control group. On the 56th day, significant differences were only observed in the Glutathione S-transferase enzyme (GST) activity and 8-OHdG content at 1.0 mg/kg compared to those in the control group due to the degradation of sulfoxaflor. This indicated that the risk of sulfoxaflor to earthworms was reduced because it was easily degraded in soil. However, because sulfoxaflor is a super toxic pollutant to earthworms, high concentrations of sulfoxaflor should not be released into the soil environment.

## 1. Introduction

According to annual global reports, crop damage caused by insect pests such as aphids has become increasingly widespread [1,2]. Sulfoxaflor, 1-(6-trifluor-methylpyridin-3-yl)ethyl)] (methyl)-oxido-l^4^-sulfanylidenec-yanamide, is used to efficiently control sap-feeding insect pests [3,4,5]. The study reported by Cutler et al. [6] proved that the pharmacological effect of sulfoxaflor on nicotinic acetylcholine receptors in aphids is consistent with that of imidacloprid. Furthermore, sulfoxaflor, a fourth-generation neonicotinoid, acts mainly on the nicotinic acetylcholine receptors (nAChRs) of the pest central nervous system, causing the pests to stop eating and eventually die [7]. However, whereas some neonicotinoids are banned in certain areas due to their increasingly negative effects, the demand for sulfoxaflor production is increasing [8] and it has been investigated for commercial development [4].

The impact of synthetic pesticides on the environment, nontarget organisms and human health has received increasing attention [7,9]. As one of the novel neonicotinoid insecticides, sulfoxaflor is slightly toxic to *Chrysoperla carnea* (Stephens) (Neuroptera: Chrysopidae) and the L_4_ larvae of *Adalia bipunctata* (L.) (Coleoptera: Coccinellidae) [7,9,10]. Furthermore, sulfoxaflor is mainly applied to control pests of important crops, such as rice, vegetables, citrus and tea trees; in this way, it is released into the soil environment. Nevertheless, there is no current research focus on hazard assessment regarding soil animals, and relatively few studies on soil ecotoxicology have been reported. Accordingly, available information about sulfoxaflor’s effects on soil organisms needs to be increased.

Earthworms are widely distributed in soil [11,12]. There is much evidence to prove the importance of earthworms in ecosystem functioning [13,14,15]. In previous research, Wang et al. [11] used a filter paper method to determine the toxicity of neonicotinoids to earthworms and found that compared with other pesticides, nicotinic insecticides showed higher toxicity to earthworms. The earthworm is a common model species for evaluating the toxic effects of chemicals on soil ecosystems because earthworms are easy to grow in the laboratory and very sensitive to most types of toxic substances [16,17,18].

Due to the combination of variations in environmental media and pollutant degradation rates, nominal concentrations are different from the concentrations that can cause toxic effects in an organism [19,20]. Because the concentration of a pollutant changes during the exposure period, it is necessary to study the concentration change trend. Accordingly, the use of effective analytical methods to determine the effective concentration of pollutants is beneficial for the evaluation of pollutant toxicity.

Consequently, the potential hazard to earthworms of exposure to sulfoxaflor was evaluated in this study. In addition, high-performance liquid chromatography-tandem mass spectrometry (HPLC-MS/MS) was used to determine the sulfoxaflor concentration in artificial soil. This study assessed the effects of sulfoxaflor on earthworms. Our finding offers theoretical data on the effects of sulfoxaflor on soil ecology. Meanwhile it is conducive to a more comprehensive risk assessment of sulfoxaflor and is beneficial to human health, environmental safety and the safe and reasonable use of neonicitinoids, specifically sulfoxaflor.

## 2. Materials and Methods 

### 2.1. Materials

Sulfoxaflor (98% purity) was purchased from Alta Scientific First Standard^®^ (Alta Scientific Co., Ltd., Tianjin, China). The chemical structural formula of sulfoxaflor is listed in Figure S1. 

The earthworms (*Eisenia fetida*) were bought from a Qingdao earthworm farm. The earthworms were healthy, with developing clitellum, and were size- and weight-matched. The earthworm body weights were approximately 310–350 mg. Before the experiments, the newly acquired earthworms were habituated to the test conditions for 2 weeks.

Artificial soil was prepared by mixing sphagnum peat moss, kaolin clay and industrial sand at a mixing ratio of 1:2:7 [21].

### 2.2. Acute Toxicity Test

The acute toxicity of sulfoxaflor to earthworms was determined using a filter paper contact test and an artificial soil test [21].

Filter paper contact treatment: the concentration gradient of sulfoxaflor consisted of 13.28, 6.64, 3.32, 1.66, 0.83, 0.415, 0.208, 0.104, 0.0525 and 0.026 µg/cm^2^. Appropriately sized filter paper was placed in a prepared glass tube and evenly wetted with 1 mL of sulfoxaflor solution or methanol solution (control) to ensure uniform distribution of the compound. After the methanol had evaporated, the filter paper was moistened with 1 mL of deionized water, and a mature and robust earthworm was placed in the tube. Each treatment was repeated ten times. All glass tubes were then placed in the dark for 48 h, and the earthworm mortality was observed.

Artificial soil treatment: The concentrations of sulfoxaflor were 0, 0.5, 1.0, 2.0, 4.0, 6.0, 8.0, 10.0, 12.0, 14.0 and 16.0 mg/kg. First, 1 mL of sulfoxaflor solution with a corresponding concentration was mixed with 50 g of artificial soil. The sample was thoroughly mixed in a 1000 mL glass beaker to ensure that the test system was uniform. In addition, 700 g of artificial soil was added to the beaker and mixed fully after the methanol evaporated. The fractional water content of the soil was 35%. Next, ten healthy earthworms were added to the beaker. There were five repeated beakers for each concentration. The beakers were placed in a culture room at 20 ± 1 °C. The earthworm mortality was examined over 14 d.

### 2.3. Subchronic Toxicity

According to a previous study of Xu et al. [22], the initial residual amount of sulfoxaflor in different soils ranges from 0.084–0.257 mg/kg. The acute toxicity test showed that the maximum concentration with no observable effect was 1.0 mg/kg; consequently, 0, 0.1, 0.5 and 1.0 mg/kg were used as test concentrations. In the 1000 mL glass beaker, these sulfoxaflor concentrations were added to 750 g of artificial soil, followed by thorough stirring to ensure methanol volatility. Next, 20 earthworms that had been gut-cleaned were placed in each beaker. There were five repeated beakers per concentration. The beakers were placed in a culture room at a temperature of 20 ± 1 °C and a light/dark period of 12/12. Then, three earthworms and 5 g artificial soil were randomly sampled form each beaker to analyze the variation of different biomarkers in the earthworm and the residual amount of sulfoxaflor in the soil on the 2nd, 7th, 14th, 28th, 42nd and 56th d. The earthworms were removed from the beaker 12 h before the biomarkers’ detection experiment and were rinsed with distilled water to remove surface soil. Then, they were placed in petri dishes covered with wet filter paper to void the gut contents.

### 2.4. Determination of Effective Concentrations

A 2.5 g aliquot of soil was obtained from previously prepared soil and placed in a 50 mL centrifuge tube. Then, 2.5 mL of deionized water was added and sulfoxaflor was extracted by 5 mL of acetonitrile. The tubes were vibrated for 5 min at 2500 rpm. Next, 2 g of MgSO_4_ and 0.5 g of NaCl were added to these centrifuge tubes, which were immediately oscillated for 5 min on a vortex mixer to prevent caking. To stratify the sample, it was centrifuged for 5 min at 4000 rpm. Eventually, to prepare the sample for HPLC-MS/MS, the supernatant was filtered through a 0.22 μm nylon syringe filter.

The extraction of sulfoxaflor was based on the use of the HPLC system. A Hypersil GOLD C_18_ column (Thermo, 2.1 × 100 mm, 3 µm, San José, CA, USA) was applied with a 10 μL injection volume. Acetonitrile (A) and 0.1% formic acid in water (B) were set to the mobile phases. The flow rate was 0.3 mL/min, and the mobile phase concentration was set as follows: 0–0.5 min, 30% A, 0.5–1.0 min, 30–95% A, 1.0–6.0 min, 95% A, 6.0–8.0 min, 95–30% A, 8.0–10.0 min, 30% A.

The multiple reaction monitoring (MRM) mode with the positive electrospray ionization (ESI^+^) was used in the present study. The capillary temperature was 350 °C, and the capillary voltage was 3.0 kV. The quantitative ion pair was 278/174 (m/z) with a collision energy of 8 eV. The qualitative ion pair was 278/154 (m/z) with a collision energy of 30 eV. The retention time of sulfoxaflor was 2.4 min.

The linearity, recovery and LOQ (Limit of Quantitation) were used to validate the residue analysis method. 

Based on the experimental concentrations, the sulfoxaflor solutions were added into the soil matrix extracting solutions at concentrations of 0.010, 0.020, 0.10, 0.20, 1.0 and 2.0 mg/kg. Then, the responses of the compounds in matrix solutions at different concentrations were detected using the residue analysis method. The calibration curve was obtained using the peak area and the matrix concentrations.

Recovery experiments were performed at three concentrations with five replicates. Sulfoxaflor solutions were added into the soil matrixes at concentrations of 0.010, 0.10 and 2.0 mg/kg. Sulfoxaflor was extracted and detected using the above method. The recovery rate was determined according to the ratio between the detected concentrations and nominal concentrations. 

The LOQ was determined according to the signal-to-noise ratio and defined at S/N ratios of 10.

### 2.5. Measurement of the ·OH^−^ Content

To determine the OH^−^ content, one earthworm was randomly selected from each beaker (five per concentration), respectively. The ·OH^−^ content was measured using the ·OH^−^ Assay Kit from Jiancheng Bioengineering Institute (Nanjing, China). The detection principle of the kit was based on the Fenton reaction. The amount of H_2_O_2_ was proportional to the amount of ·OH^−^ produced by the reaction. When an electron acceptor was added, the Griess reagent turned red in proportion to the amount of ·OH^−^. According to the instruction, there were four treatments: standard blank tube (tube 1), standard tube (tube 2), control tube (tube 3), test tube (tube 4). After adding 0.4, 0.2, 0.2 mL of distilled water to tube 1, 2, 3, respectively, the 0.03% H_2_O_2_ standard application liquid of 0.2 mL was added to the tube 2. Then, 0.2 mL the substrate application solution was added to tube 3 and tube 4. After adding 0.2 mL of the sample solution to the tube 4, 0.4 mL of the reagent three application solution was added to each of the four tubes. Next to it, it was necessary that the 2 mL chromogenic agent was immediately added to the tube after the reagent mixture reacted 1 min accurately in the tube. After adding the relative dose of the reaction liquid in turn, the absorbance was determined at 550 nm according to the protocol.

### 2.6. Measurement of Enzyme Activity

Before detecting the indicators and to determine antioxidant enzyme activities and thiobarbituric acid reactive substances (TBARS) content, one earthworm was randomly selected from each beaker (five per concentration), respectively. The enzyme solution of the earthworms that had voided the gut contents was extracted using a potassium phosphate buffer with pH 7.8 (1:10, w/v) in each treatment. The samples were placed on ice during the entire experiment, and the protein content was determined before measuring other indicators [23].

The superoxide dismutase (SOD) activity was measured in accordance with the method of Song et al. [24]. According to the experimental principle, SOD activity can be obtained by detecting the amount needed to induce nitroblue tetrazolium chloride (NBT) reduction by measuring the absorbance at 560 nm. The amount of enzyme activity required to inhibit the photochemical reduction of NBT is the enzyme activity unit (U).

Catalase (CAT) activity was determined using the method of Liu et al. [23]. Upon the decomposition of H_2_O_2_ by CAT, the gradual decrease in the absorbance within 1 min was monitored at 250 nm. A decrease of 0.1 OD per min is one enzyme activity unit (U).

Glutathione S-transferase enzyme (GST) activity was measured in accordance with the method reported by Liu et al. [23]. This method monitors the increase in absorbance for 3 min at 340 nm. The amount of GST that leads to the combination of glutathione (GSH) and 1-chloro-2,4-dinitro-benzene (CDNB) is the enzyme activity unit (U).

### 2.7. Measurement of TBARS Content

The TBARS, mainly malonaldehyde (MDA), is an important indicator to reflect the level of lipid peroxidation and is determined by monitoring the reaction between MDA and thiobarbituric acid (TBA) [25]. After heating at 95 °C in a water bath for 1 h, the absorbance of the red reaction product was detected at 532 nm [26]. The result was expressed as nmol TBARS mg^−1^ Protein.

### 2.8. Measurement of 8-OHdG Content

To determine the 8-OHdG content, one earthworm was randomly selected from each beaker (five per concentration), respectively. The content of 8-hydroxy-2′-deoxyguanosine (8-OHdG) was measured in the light of the method supplied by the 8-OHdG ELISA kit from the Jiancheng Bioengineering Institute (Nanjing, China). The sample contained concentrated washing liquid, enzyme labelled reagent, sample diluent, stop solution, standard solution, standard diluent, chromogenic agent A and chromogenic agent B. According to the instructions, there were blank holes, standard holes and sample holes that had been measured in the test. Firstly, 50 μL of the standard solution was accurately added to the enzyme-coated plate and 40 μL of the sample diluent was added to the sample well, followed by 10 μL the sample solution. After the enzyme labelled plate was sealed with the closure membrane, the plate was incubated at 37 °C for 30 min. Then the membrane was carefully discarded, and the liquid discarded. Each hole was filled with washing liquid which had been diluted by distilled water 30 times. The washing liquid was discarded after 30 s repeated 5 times and patted dry. Next to it, 50 μL enzyme labelled reagent was added to each well, except for blank wells. After repeating the process of breeding and washing, the chromogenic agent A and the chromogenic agent B were added in each hole. Finally, take blank hole as zero, the absorbance of each hole under the condition of 450 nm was measured.

### 2.9. Statistical Analysis

SPSS software (version 22.0, SPSS Inc., Chicago, IL, USA) was used to analyze the data which were expressed as the mean ± SD. Two-way analysis of variance (ANOVA) was used to measure the effects of concentration, exposure time, and their interaction on biomarkers. A significant difference was indicated by *p* < 0.05, which was ascertained by using the least significant difference (LSD) test.

## 3. Results and Discussion

### 3.1. Acute Toxicity of Sulfoxaflor on Earthworms

**Figure 1:**
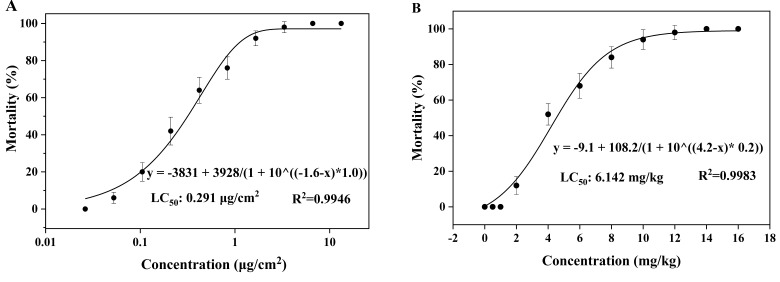
Dose-effect curves of the contact filter paper test (**A**) and the acute artificial soil test (**B**) after exposure to sulfoxaflor.

As shown in Figure 1, the mortality rate of the earthworms in both the filter paper treatment and artificial soil treatment increased with increasing sulfoxaflor concentrations during the whole exposure period. The LC_50_ values for sulfoxaflor in the contact filter paper test and the artificial soil test were 0.291 µg/cm^2^ and 6.142 mg/kg, respectively, which demonstrated that sulfoxaflor is a supertoxic pollutant to earthworms [26]. In comparison to another neonicotinoid insecticide, imidaclothiz, the 7 d and 14 d LC_50_ values for earthworms were found to be 0.86 and 2.15 mg/kg, respectively [27]. Chen et al. [28] supplemented the toxicity study of imidacloprid and found that the LC_50_ values ranged from 5.46 to 12.58 mg/L and from 2.34 to 4.03 mg/kg in the acute contact filter paper test and artificial soil test, respectively. These results show that neonicotinoid insecticides, such as sulfoxaflor, imidaclothiz, and imidacloprid, are highly toxic to earthworms. The toxicity of sulfoxaflor to invertebrates in soil requires attention, but on the other hand, compared to imidaclothiz and imidacloprid, sulfoxaflor is less toxic to earthworms.

### 3.2. Effective Concentrations of Sulfoxaflor

In this study, the detection method of sulfoxaflor had good linearity in the soil matrix. The calibration equation and R^2^ (correlation coefficients) of sulfoxaflor in soil was y = 100,216,755 x + 409,773 and 0.9997, respectively. The recoveries were 97.8–98.5% with the relative standard deviation (RSD) of 2.2–3.2% at three spiked concentrations of 0.010, 0.10 and 2.0 mg/kg. The LOQ of sulfoxaflor was 0.010 mg/kg. The specific values are listed in Table S1. These results indicate that this detection method has high sensitivity and accuracy and could be used to detect sulfoxaflor in soil matrix. 

**Figure 2:**
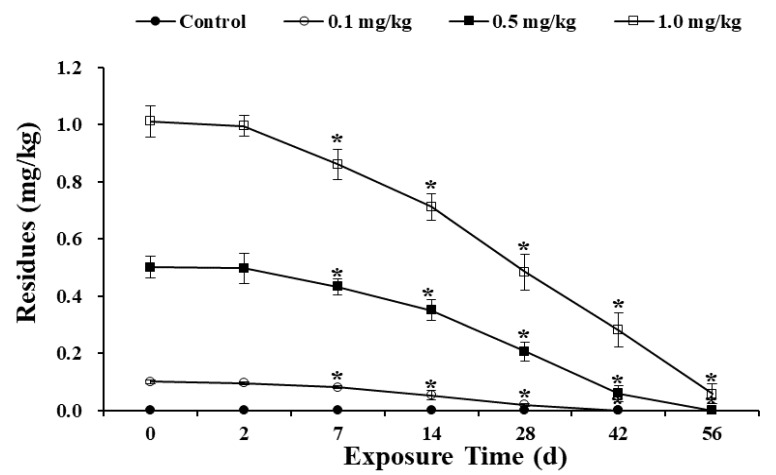
Residues of sulfoxaflor in artificial soil during the exposure period. Bars are the means ± SD of five replicates. Asterisks indicate the significant differences (*p* < 0.05) between the exposure time.

The change in residual sulfoxaflor in the soil over the course of the exposure time is shown in Figure 2. In the control group, no residual sulfoxaflor was found over the whole period. Overall, increasing exposure time led to a decreasing amount of residual sulfoxaflor in the soil throughout the exposure period. The sulfoxaflor in the 0.1 mg/kg treatment group was completely degraded on day 42. On the 56th d, the sulfoxaflor in the 0.5 mg/kg treatment group was completely degraded, while the sulfoxaflor in the 1.0 mg/kg group was degraded by 94.27%. Among the different treatment groups, the half-life of sulfoxaflor ranged from 12.0–15.4 d, and the degradation rate in the artificial soil ranged from 0.002–0.017 mg/(kg·d). The half-life of sulfoxaflor was different at different concentrations, and the degradation rate in the high-concentration treatment group was higher. According to previous research, the half-lives of other neonicotinoid insecticides such as imidaclothiz and dinotefuran were as long as 57.8 and 100 d, respectively [27,29]. Compared with them, sulfoxaflor is more easily degraded in soil and has significant advantages. Based on acute test results, sulfoxaflor is less toxic to earthworms compared with imidaclothiz, imidacloprid and dinotefuran. Therefore, it is recommended to use sulfoxaflor in actual production.

In a previous study, the half-lives of sulfoxaflor in three soil samples from Shandong, Henan and Zhejiang were 6.3, 7.2 and 1.5 d [22], respectively. This study result presents a certain discrepancy with the findings of previous research, which may be due to the different soil types affecting the degradation rate of pesticides. Specifically, it may be because natural soils have more numbers and types of microorganisms that degrade pesticides, and natural soils contain more nutrients than artificial soils to facilitate microbial reproduction. In addition, the field soil environment is more suitable for microbial survival than the laboratory environment. But on the other hand, these results show that sulfoxaflor is easily degraded in soil. 

### 3.3. Two-Way ANOVA Results

As shown as in Table 1, the concentrations, exposure time or their interaction have significant effects on the biomarkers in earthworms (*p* < 0.05). The results showed that both concentration and exposure time could affect the toxicity of sulfoxaflor to earthworms. It may be due to earthworms accumulating more and more sulfoxaflor with the increase of concentrations and exposure time, finally leading to a stronger toxic effect. Similar results have been found by Liu et al. [29] when they studied the toxicity of dinotefuran on earthworms. Additionally, Wang et al. [30] also reported a similar result when they studied the toxicity of imidacloprid on earthworms. In their research, all biomarkers were significantly influenced by dose, exposure time and their interaction. 

### 3.4. Effects of Sulfoxaflor on the ·OH^−^ Content

**Figure 3:**
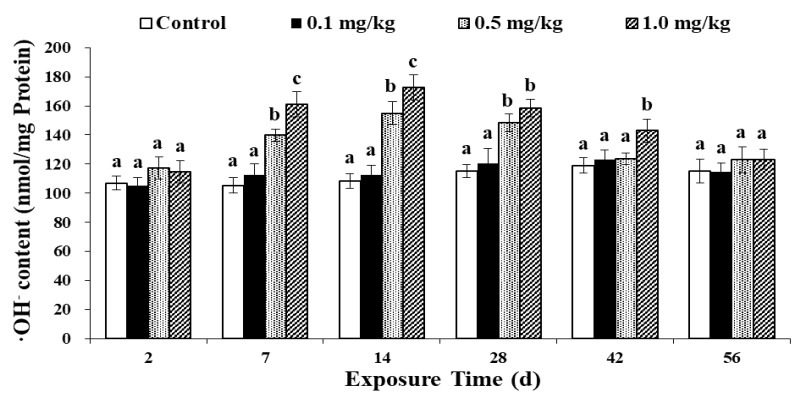
Effects of sulfoxaflor on the ·OH^−^ content in earthworms. Bars are the means ± SD of five replicates. Different letters indicate significant differences (*p* < 0.05) between treatments.

When an organism is exposed to toxic chemicals, it produces and accumulates reactive oxygen species (ROS), which play extremely important roles in redox homeostasis and cell antioxidant signal transduction, and excess ROS can harm proteins, lipids and nucleic acids [31,32,33]. ROS is the general name given to various molecules, including superoxide anion (O^2−^), hydroxyl radical (OH^−^), hydrogen peroxide (H_2_O_2_), singlet oxygen and other free radicals [26,34]. ROS are mainly produced by the transfer of single electrons to oxygen in the mitochondrial respiratory chain [35,36]. This study examined the change in ·OH^−^ content after exposure to sulfoxaflor (Figure 3). Throughout the exposure period, comparing the control group with the 0.1 mg/kg treatment group, no significant change in the ·OH^−^ content was found (*p* > 0.05). However, on days 7, 14 and 28, compared to that in the control group, the OH^−^ content in these two treatment groups increased significantly (*p* < 0.05). In the 0.5 mg/kg and 1.0 mg/kg treatment groups, the OH^−^ content reached the highest level on the 14th day and then decreased gradually to the same concentration as that in the control group on day 56. Moreover, Liu et al. [29] found that the level of ROS in earthworms treated with another neonicotinoid insecticide dinotefuran increased significantly, proving that dinotefuran causes oxidative stress. These results are similar to the findings of Zhang et al. [33], who researched the ecotoxicological effect of imidacloprid on earthworms. The ROS level in earthworms increased significantly with increasing treatment doses (0.66, 2, and 4 mg/kg) in their assay. Therefore, the significant increase in the ·OH^−^ content in the treatment groups indicates that sulfoxaflor can cause earthworm cell damage. In this study because sulfoxaflor was easily degraded in soil, the concentration of sulfoxaflor rapidly decreased over time. The toxic effect therefore decreased, and the antioxidant enzymes could deal with the excess ROS. As a result, the concentration of ·OH^−^ was restored to the control level at the end of exposure.

### 3.5. Effects of Sulfoxaflor on Antioxidant Enzymes Activities

**Figure 4:**
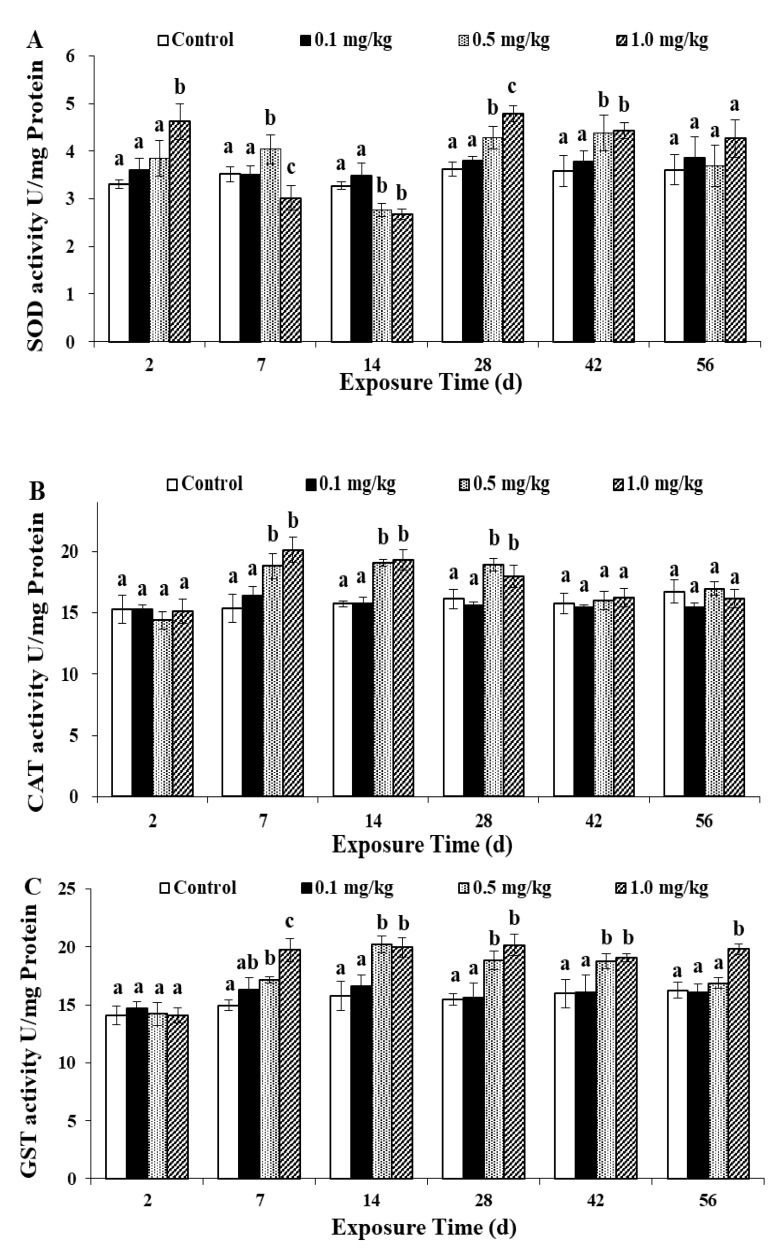
Effects of sulfoxaflor on the superoxide dismutase (SOD) (**A**), catalase (CAT) (**B**) and glutathione S-transferase enzyme (GST) (**C**) activities in earthworms. Bars are the means ± SD of five replicates. Different letters indicate significant differences (*p* < 0.05) between treatments.

Excessive ROS can cause oxidative stress in organisms [27]. The cellular antioxidant enzymes can represent indirect physiological indicators of oxidative stress in the body when stimulated by pollutants [37]. Therefore, researchers pay substantial attention to these important biomarkers [30,33]. The antioxidant enzymes detected in this study were SOD, CAT and GST. The results are shown in Figure 4.

The SOD level maintains a dynamic balance to meet the needs of the body and eliminate excess oxygen under normal physiological conditions [17,29,38]. When an organism is subjected to oxidative stress, SOD can convert O_2_^−^ into H_2_O_2_ through a dismutation reaction [33,39]. In this assay, with increasing sulfoxaflor concentrations, the SOD activity increased on the 2nd day, showing a dose-response relationship (Figure 4A). Throughout the exposure period, the difference between the control group and the 0.1 mg/kg treatment group was not significant (*p* > 0.05). However, the SOD activity in the 1.0 mg/kg and 0.5 mg/kg treatments increased significantly (*p* < 0.05) on days 2 and 7, respectively. The SOD activities in the 0.5 and 1.0 mg/kg treatments decreased significantly (*p* < 0.05) on day 14, while they increased significantly (*p* < 0.05) on days 28 and 42 compared with that in the control. When the exposure was terminated on day 56, the difference in SOD activity between the treatment and control was not significant (*p* > 0.05). In this study, on the 2nd and 7th day, earthworms were affected by the toxicity of pesticides to produce oxidative stress in vivo. In order to eliminate excessive ROS, SOD activity increased. By 14 days, the earthworms fed on the soil continuously and accumulated sulfoxaflor in the body, which exceeded the earthworm’s own regulating ability and inhibited the activity of SOD. However, 28 days later, earthworms gradually adapted to the environment, and SOD activity increased to eliminate excessive active oxygen. By the end of the experiment, SOD activity had returned to the control level.

CAT typically can transform the SOD free radical product H_2_O_2_ into harmless water and oxygen, thereby eliminating the toxicity of H_2_O_2_ to the organism [26,40]. Changes in CAT activity are shown in Figure 4B. In this experiment, similar to the results for SOD activity, there was no significant difference in CAT activity between the 0.1 mg/kg treatment group and control group (*p* > 0.05). Nevertheless, the CAT activities in the 0.5 mg/kg treatment group and 1.0 mg/kg treatment group increased significantly from day 7 to day 28 compared with that in the control group (*p* < 0.05). The CAT activities in all treatments were similar to that of the control group on the 42nd day and on the 56th day (*p* > 0.05). In this study, compared with CAT, SOD had a stronger response to sulfoxaflor-induced oxidative stress. On the whole, CAT activity increased with the increase of SOD content on the 2–7th day. After the 7th day, SOD activity was inhibited, but H_2_O_2_ produced by SOD scavenging excess ROS accumulated in earthworm, and the CAT activity for scavenging excess H_2_O_2_ continued to increase. On the 42nd day, the CAT activity had returned to the control level.

According to the SOD and CAT activity results, no oxidative damage occurred in earthworm cells in the 0.1 mg/kg treatment. In the early stage of exposure, the activities of SOD and CAT increased, and the oxidative damage caused by sulfoxaflor could be prevented. Specifically, the SOD activity of the 1.0 mg/kg treatment group increased on the 2nd day; it increased on the 7th day of the 0.5 mg/kg treatment group, while the CAT activity of the 0.5 and 1.0 mg/kg treatment groups increased. The strong toxic effect of sulfoxaflor may be due to inhibited and reduced SOD activity. Finally, the toxic effect of sulfoxaflor decreased with decreasing concentration, which caused the SOD and CAT activities to return to the control level. Feng et al. [31] found that the activities of SOD and CAT were inhibited significantly in a 3 mg/kg treatment group compared with the control group by studying the oxidative stress of earthworms after exposure to thiacloprid. In their study, the activities of SOD and CAT were restored to the control level when exposure was terminated, which was similar to our experimental results, suggesting that earthworms have the ability to recover from the stress of thiacloprid. Therefore, we can infer that sulfoxaflor poses a danger to earthworms. Because sulfoxaflor is easily degraded in soil, decreasing amounts of pesticide remain in the soil over time. The antioxidant enzyme system in earthworms exposed to sulfoxaflor is able to remove excess ROS to protect against oxidative damage, decreasing the toxic effect.

GST is one of the most important detoxification enzymes; it can address various exogenous compounds such as pesticides and inhibit the toxicity of ROS [20,35]. GST activity (Figure 4C) showed an overall upward trend. When the sulfoxaflor concentration was increased, the activity of GST increased gradually. Furthermore, no significant difference in GST activity between the control group and the 0.1 mg/kg treatment group was found (*p* > 0.05). A significant improvement in GST activity was observed at doses of 0.5 and 1.0 mg/kg sulfoxaflor from day 7 to day 42 (*p* < 0.05) compared to the control group. At the end of the experiment, the GST level of 1.0 mg/kg treatment group still did not return to the control level. Based on these results, we can infer that the toxicity of sulfoxaflor can increase ROS in earthworm cells and promote the activity of GST. Zhang et al. [27] found a similar result which studied the oxidative stress of imidaclothiz on earthworms. In accordance with the essay, the GST activity was significantly increased in the 0.5 mg/kg treatment compared to the control on days 35, 42 and 56. Wang et al. [30] also found that GST activity was induced by imidacloprid during exposure. These results show that GST activity is an effective biomarker to evaluate pollutant toxicity.

### 3.6. Oxidative Damage Effects in Earthworms Induced by Sulfoxaflor

**Figure 5:**
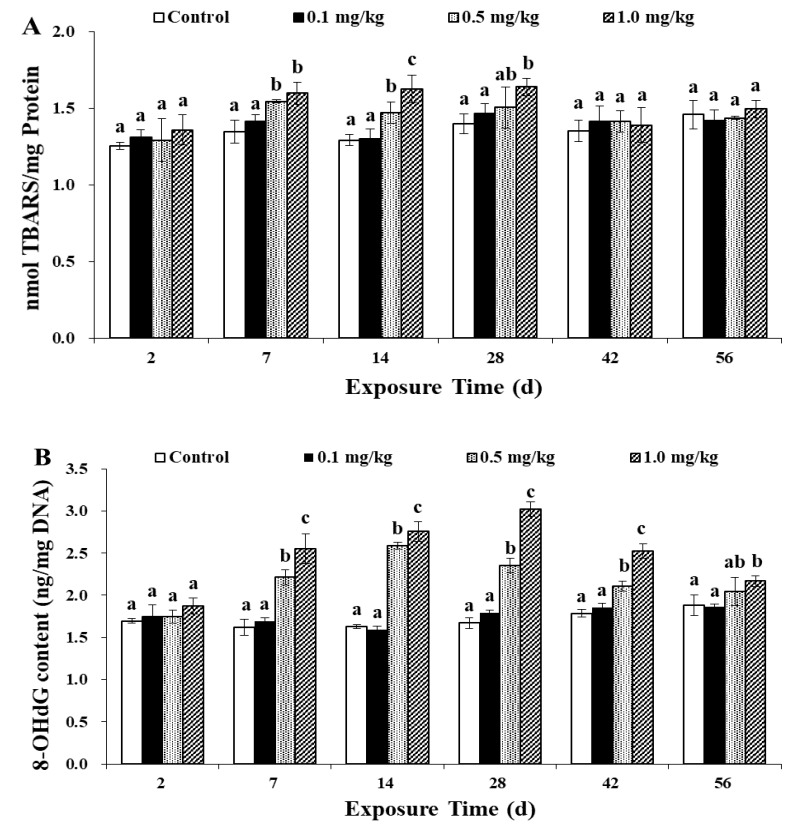
Effects of sulfoxaflor on the thiobarbituric acid reactive substances (TBARS) (**A**) and 8-OHdG (**B**) content in earthworms. Bars are the means ± SD of five replicates. Different letters indicate significant differences (*p* < 0.05) between treatments.

Excess ROS exceeds the scavenging ability of antioxidant enzymes, causing oxidative damage in cells [20,23]. The results of this study are shown in Figure 5.

TBARS were cytotoxic final products of cell lipid peroxidation; content could reflect whether the body is subjected to lipid peroxidation [25,41,42]. Moreover, the TBARS content could illuminate whether external pressure causes cell damage [30,35]. In the current study (Figure 5A), compared to that in the control group, no significant difference was observed in the TBARS content at doses of 0.1 mg/kg sulfoxaflor during the exposure period. On days 7, 14 and 28, compared with that in the control group, the TBARS content at the doses of 0.5 and 1.0 mg/kg sulfoxaflor increased significantly (*p* < 0.05). With increasing sulfoxaflor concentration, the TBARS content also increased gradually from day 7 to day 28. On days 42 and 56, there were no significant differences in TBARS content between the treatment and control (*p* > 0.05). That toxic pollutants could induce lipid peroxidation and cell damage in earthworms was also reported in the article, which studied the toxicity of BDE209 and Pb to earthworms [17]. In this study, the MDA content increased significantly in all BDE209-Pb treatments (*p* < 0.05) compared to that in the control group. The present result was also consistent with the research reported by Ye et al. [12] and Markad et al. [42]. All these studies show that increased TBARS content could be an indirect biomarker of oxidative damage induced by pollutants. Therefore, we can deduce that sulfoxaflor can cause cytotoxicity to earthworms. 

8-OHdG is one of the important indicators reflecting DNA oxidative damage, and a large number of studies indicate that 8-OHdG can be used as a biomarker for DNA oxidative damage by endogenous and exogenous factors [43,44,45]. As shown in Figure 5B, from day 7 to day 28, the content of 8-OHdG showed an upward trend. The content of 8-OHdG increased with increasing concentrations of sulfoxaflor and showed a dose-response relationship. Significant increases in 8-OHdG content were found on days 7, 14, 28, 42 and 56 at doses of 0.5 and 1.0 mg/kg sulfoxaflor (*p* < 0.05). Throughout the exposure period, there were no significant differences in 8-OHdG content between the 0.1 mg/kg treatment group and the control group (*p* > 0.05). In this study, at the end of the exposure the 8-OHdG content of the 1.0 mg/kg treatment group still did not return to the control level. This result may be because the exposure time was not long enough, the content of 8-OHdG content was too late to return to the control level, or it may be due to the toxicity of sulfoxaflor to earthworms, which caused irreversible damage to the DNA of earthworm. Topal et al. [45] found similar results by researching the effect of imidacloprid on the 8-OHdG content in rainbow trout. These findings indicated that 8-OHdG can be used as a sensitive biomarker for DNA damage, and the increased 8-OHdG content indicates that the sulfoxaflor may have caused genotoxicity to earthworms.

In the present study, although sulfoxaflor caused lipid peroxidation and DNA damage in earthworms at the early stage of exposure, the toxic effects were alleviated at the end of exposure. The reason may be due to the degradation of sulfoxaflor in artificial soil at the end of the exposure period. As sulfoxaflor was easily degraded in soil, its risk to earthworms was reduced. However, high concentrations of sulfoxaflor should not be released into the soil environment as it is a super toxic pollutant to earthworms.

## 4. Conclusions

In this study, a variety of biomarkers were used to assess the potential danger of sulfoxaflor to earthworms. The main conclusions of this study include the following:

- Sulfoxaflor is a supertoxic pollutant to earthworms with the LC_50_ values of 0.291 µg/cm^2^ and 6.142 mg/kg, respectively.
- Sulfoxaflor was degraded quickly in the artificial soil with the degradation rate of 0.002–0.017 mg/(kg·d) and a half-life of 12.0–15.4 d at different concentrations.
- The changes in the activity or content of each biomarker in 0.5 mg/kg and 1.0 mg/kg treatment demonstrated that sulfoxaflor caused oxidative damage at the early stage of exposure. At the end of exposure, the toxic effects were reduced due to the degradation of sulfoxaflor.
- 8-OHdG is a sensitive indicator to monitor the toxicity of sulfoxaflor in earthworms.
- The risk of sulfoxaflor to earthworms was reduced because of it was easily degraded in soil. However, high concentrations of sulfoxaflor should not be released into the soil environment as it is a super toxic pollutant to earthworms.

## Figures and Tables

**Table 1:** Two-way ANOVA result for biomarkers in earthworms exposed to sulfoxaflor.

Biomarkers	Dose			Time			Dose × Time		
	df	F	*p*	df	F	*p*	df	F	*p*
OH^−^	3	98.38	0.000 *	5	25.22	0.000 *	15	8.92	0.000 *
SOD	3	10.61	0.000 *	5	25.74	0.000 *	15	7.62	0.000 *
CAT	3	29.98	0.000 *	5	22.76	0.000 *	15	6.69	0.000 *
GST	3	60.72	0.000 *	5	30.59	0.000 *	15	4.77	0.000 *
TBARS	3	16.13	0.000 *	5	10.09	0.000 *	15	2.13	0.025 *
8-OHdG	3	307.90	0.000 *	5	36.28	0.000 *	15	22.65	0.000 *

df: Degree of freedom. * Cases where the concentration or the duration of exposure had a significant effect (*p* < 0.05). SOD: Superoxide dismutase, CAT: Catalase, GST: Glutathione S-transferase, TBARS: thiobarbituric acid reactive substances.

## Supplementary Materials

The following are available online at https://www.mdpi.com/1660-4601/17/5/1740/s1, Figure S1: The chemical structural formula of sulfoxaflor. The image was completed using the ChemBioDraw software (version 11.0, CambridgeSoft Corporation, Cambridge, MA, USA), Table S1: The recovery (%) and RSD (%) of sulfoxaflor in soil at three spiked levels.
