# Supplementary material for: The Toxic Effects of Sulfoxaflor Induced in Earthworms (Eisenia fetida) under Effective Concentrations
Source: Int J Environ Res Public Health. 2020 Mar 7;17(5):1740. doi: 10.3390/ijerph17051740 (PMC7084856; doi:10.3390/ijerph17051740)
Supplement: Supplementary file 1 [file ijerph-17-01740-s001.pdf]

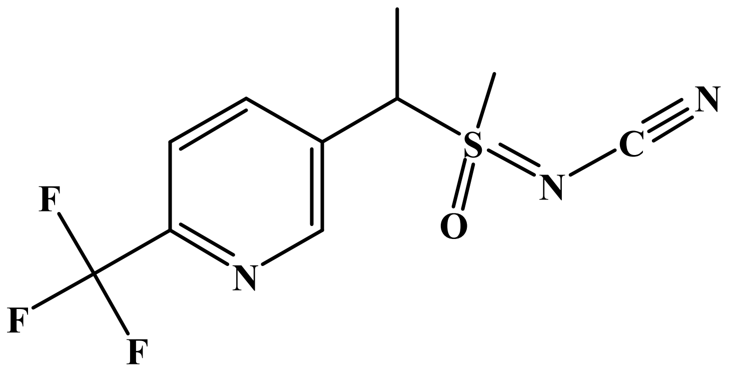

Figure S1. The chemical structural formula of sulfoxaflor. The image was completed using the ChemBioDraw software (version 11.0).

Table S1. The recovery (%) and RSD (%) of sulfoxaflor in soil at three spiked levels.

Matrixes	Spiked level (mg/kg)	sulfoxaflor	
		Recovery	RSD
Soil	0.010	98.0	2.7
	0.10	97.8	3.2
	2.0	98.5	2.2
